# “Too much, too late”: data on stillbirths to improve interpretation of caesarean section rates

**DOI:** 10.2471/BLT.21.287539

**Published:** 2022-02-28

**Authors:** Siem Zethof, Aliki Christou, Lenka Benova, Jos van Roosmalen, Thomas van den Akker

**Affiliations:** a Department of Obstetrics, Leiden University Medical Centre, Albinusdreef 2, 2333 ZA, Leiden, Netherlands.; b Department of Public Health, Institute of Tropical Medicine, Antwerp, Belgium.; c Athena Institute, VU Amsterdam, Amsterdam, Netherlands.

Babies born from 28 weeks gestation onward have a considerable chance of survival. The World Health Organization defines the death of a fetus beyond this gestational age and before birth as a stillbirth. Like many deaths that are largely preventable, the 2 million stillbirths occurring worldwide every year are inequitably distributed.^1^ Low- and middle-income countries carry the largest burden, accounting for up to 98% of stillbirths. Of the estimated 1 966 000 stillbirths per year worldwide, only 38 000 occur in high-income countries.^1^ In the past decade, awareness of the high burden of stillbirths has increased, as the global health community has highlighted their impact on the lives of affected parents and communities.^2^

Stillbirth rates are increasingly regarded as an important indicator of the quality of maternity care.^1^^,^^3^ Nevertheless, many low- and middle-income countries lack accurate and timely data about stillbirths because of limited coverage of civil registration and vital statistics. Instead, national – and therefore global – estimations of stillbirths rely, for the most part, on population-based household surveys such as Demographic and Health Surveys (DHS) and Multiple Indicator Cluster Surveys.^1^ To date, these surveys do not capture data pertaining to antenatal and intrapartum care received by women reporting stillbirths, including data regarding mode of birth. Lack of such data leaves an important knowledge gap regarding the impact of interventions on maternal and newborn outcomes.

Recent revisions to the DHS questionnaire (DHS-8) have led the DHS programme to adopt a full pregnancy history module, enabling capture of data on health-care use during pregnancy, as well as mode of birth, for all births.^4^ In the following discussion, we argue that using data collected through the recently revised questionnaire will be critical to our understanding of caesarean section rates and practices in settings with limited data.

Performing caesarean sections either too little, too late (when access is limited) or too much, too soon (when not medically indicated) increases risks of maternal and perinatal mortality and morbidity.^5^ Caesarean sections may also be performed too much, too late, for women whose baby had already died at the time of surgery. Conducting such procedures without maternal indication exposes women to risks of surgery without saving the life of the baby. This situation is important in low-income settings, as emergency caesarean sections are associated with increased maternal risk of haemorrhage and infection, which may be fatal when there is a shortage of resources to manage these complications.^5^

Some caesarean sections may be necessary in women with intrauterine fetal death to manage severe maternal complications of pregnancy by prompting immediate birth to save a woman’s life. Such is the case of severe antepartum haemorrhage, life-threatening eclampsia and pre-eclampsia, and some cases of cephalo-pelvic disproportion. Others may have been performed in an attempt to save a baby whose heart was still heard at the start of surgery. In settings with high stillbirth rates, studies suggest that half of all fetal deaths occur during labour, many of which could have been prevented by quality intrapartum care, including emergency caesarean section.^1^ In the absence of electronic fetal heart rate monitoring or Doppler ultrasound, use of Pinard stethoscopes to diagnose stillbirth may lead to misdiagnosis and misuse of emergency caesarean section. However, many caesarean sections ending in stillbirth are not conducted for any of these reasons but rather for prolonged labour, while the fetal heart rate was either not listened to or listened to but not heard at the time of decision for surgery.^3^^,^^6^^,^^7^

Instead of performing caesarean section, alternative options for birth could be considered after fetal death has been confirmed, such as induction of labour, assisted vaginal birth and, in extreme cases, destructive operative vaginal birth. Women should be given the opportunity for an informed choice with the consequences of caesarean section for current and potential future pregnancies clearly discussed.

Currently, critical data are unavailable for pregnancies ending in stillbirth, and the exact scale and quality of practice of caesarean sections that are performed without indication or too much, too late, remains invisible, as do the consequences of those caesarean sections on maternal morbidity, including women’s mental health. Little is known about whether the quality of care during caesarean section directly affects risk of stillbirth or if neglect of women experiencing intrauterine fetal death may result in poor intrapartum management.^3^^,^^8^ However, stillbirths are seen as a sensitive indicator of substandard intrapartum care, including challenges in intrapartum monitoring, use of oxytocin, timely decision-making and documentation.^3^^,^^6^ National and local studies evaluating quality of maternity care should strive to include women experiencing stillbirth, because exclusion of such a detrimental outcome of pregnancy results in overestimating quality of care and may contribute to missed opportunities for improvement, especially in the presence of current excessive caesarean section rates associated with stillborn babies.

Global caesarean section rates have been rising for the past 30 years and are expected to increase even further, with almost 30% of births globally are predicted to take place by caesarean section in 2030.^9^ National rates range from as low as 1% to as high as 58% and considerable wealth-related inequalities exist within countries.^9^ When estimating national caesarean section rates using population-based household survey data from low- and middle-income countries, caesarean section rate is defined as the number of caesarean births per 100 live births, due to lack of data on mode of birth among women who had stillbirths.^9^

The inclusion of stillbirths in calculations of caesarean section rates may alter the prevalence of caesarean sections, particularly if the percentage of stillborn babies born by caesarean section differs from the caesarean section rate among live births. Large effects on caesarean section rates will be seen in settings with high stillbirth rates, for example in sub-Saharan Africa, where 1 in 40 pregnancies and 1 in 10 births by caesarean section were reported to end in stillbirth.^1^^,^^5^

In studies in low- and middle-income countries in South-East Asia, South America and sub-Saharan Africa, population-based caesarean section rates among stillbirths ranged from 8% (533/7053) to 16% (160/1033), depending on the definition of stillbirth, and did not differ markedly from rates among live births.^6^^,^^10^ Two facility-based studies in sub-Saharan Africa documented much higher caesarean section rates among stillbirths than live births with rates of 26% (35/135) and 43% (64/150) among stillbirths – more than double the rates of live births.^3^^,^^7^ Given the high stillbirth rates in many low- and middle-income countries, adding these hidden caesarean sections to the calculation could increase caesarean section rates substantially. To date, few studies using household survey data mention the exclusion of stillbirths as a limitation to the validity of their calculated caesarean section rates. 

We recognize that population-based household surveys are limited with regard to the information they can capture about stillbirths. Women who respond to the questionnaire might be unaware whether the child died prior to, during or after caesarean section, and asking detailed questions could aggravate their grief and pain. Additionally, we know nothing about stillbirths or caesarean sections among women who died during pregnancy, childbirth or the postpartum period, whose tragedies are not reflected in population-based surveys from self-report at all. Therefore, health-facility registers are an important potential source of stillbirth data, yet these are underutilized and concerns exist about underreporting and misclassification of stillbirths based on such registers.^3^^,^^11^ As facility-based births increase worldwide, improving quality of antenatal, intrapartum and postnatal care should be prioritized. Strengthening of facility-based data collection and documentation is an important step towards achieving such improvements. For example, performing perinatal death reviews in women with caesarean section may contribute to obtaining more robust stillbirth data, as well as to improvement of caesarean-related and childbirth care by enhancing professional learning and increasing accountability.^12^ Similarly, low cost strap-on continuous fetal heart rate monitoring devices may not only reduce perinatal mortality by detecting signs of fetal distress, but also improve identification of intrauterine fetal demise and establishing timing of death to improve the quality of death reviews. Data derived through such enhanced means may establish reliable information on all births, including indications of caesarean section and timing of surgery related to fetal heart rate monitoring in women with stillbirths, to understand the true scale of potentially unnecessary and harmful interventions.

As clinicians and maternal health researchers, we are hopeful that questionnaires capturing full pregnancy histories, such as DHS-8, will be used in future surveys. By including all pregnancies, reporting of stillbirths will increase and enhance our understanding of the associated factors. Such knowledge will also contribute to informing policy-making for health system improvements such as improving antenatal care coverage or intrapartum monitoring to prevent adverse pregnancy outcomes.^10^ In addition, the use of full pregnancy histories will lead to improved understanding of caesarean section rates among all births. In doing so, obstetric care can improve by preventing caesarean sections from happening too much, too late, and instead providing the right amount of care at the right time.
